# Delivery of DNA into Human Cells by Functionalized Lignin Nanoparticles

**DOI:** 10.3390/ma15010303

**Published:** 2022-01-01

**Authors:** Michael K. Riley, Wilfred Vermerris

**Affiliations:** 1 Graduate Program in Plant Molecular & Cellular Biology, University of Florida, Gainesville, FL 32610, USA; mike.riley350@gmail.com; 2 Department of Microbiology & Cell Science, University of Florida, Gainesville, FL 32610, USA; 3 UF Genetics Institute, University of Florida, Gainesville, FL 32610, USA; 4 Florida Center for Renewable Chemicals and Fuels, University of Florida, Gainesville, FL 32610, USA

**Keywords:** BioLignin, gene therapy, solvent exchange, thioglycolic acid

## Abstract

Lignin is an aromatic plant cell wall polymer that is generated in large quantities as a low-value by-product by the pulp and paper industry and by biorefineries that produce renewable fuels and chemicals from plant biomass. Lignin structure varies among plant species and as a function of the method used for its extraction from plant biomass. We first explored the impact of this variation on the physico-chemical properties of lignin nanoparticles (LNPs) produced via a solvent exchange procedure and then examined whether LNPs produced from industrial sources of lignin could be used as delivery vehicles for DNA. Spherical LNPs were formed from birch and wheat BioLignin™ and from poplar thioglycolic acid lignin after dissolving the lignin in tetrahydrofuran (THF) and dialyzing it against water. Dynamic light scattering indicated that the diameter of these LNPs was dependent on the initial concentration of the lignin, while electrophoretic light scattering indicated that the LNPs had a negative zeta potential, which became less negative as the diameter increased. The dynamics of LNP formation as a function of the initial lignin concentration varied as a function of the source of the lignin, as did the absolute value of the zeta potential. After coating the LNPs with cationic poly-l-lysine, an electrophoretic mobility shift assay indicated that DNA could adsorb to LNPs. Upon transfection of human A549 lung carcinoma basal epithelial cells with functionalized LNPs carrying plasmid DNA encoding the enhanced green fluorescent protein (eGFP), green foci were observed under the microscope, and the presence of eGFP in the transfected cells was confirmed by ELISA. The low cytotoxicity of these LNPs and the ability to tailor diameter and zeta potential make these LNPs of interest for future gene therapy applications.

## 1. Introduction

Nanomedicine is an emerging field that utilizes nanotechnology for diagnostic and therapeutic purposes. The small size and tunable properties of nanomaterials enable smart delivery of therapeutic agents at target sites, confer theranostic properties (simultaneous diagnosis and therapeutic action [1]) and enhance the ability to image tissues and organs in vivo [2,3]. An area of particular interest is gene therapy, which refers to methods aimed at altering gene expression in living organisms through the delivery of exogenous DNA and/or RNA. Although approved gene therapy methods currently rely on viral delivery of exogenous DNA with adeno-associated virus [4], the rapid developments in nanomedicine offer the near-term prospect of alternative delivery methods with potentially greater efficacy and/or fewer side effects.

One of the challenges associated with the use of nanomaterials as delivery vehicles is their cytotoxicity. For example, carbon nanotubes are known to cause damage from their ability to puncture cells [5]. Nanoparticles derived from natural products to which the human body is exposed in the digestive tract have the potential to be less cytotoxic [3]. The aromatic plant cell wall polymer lignin is of interest in this respect.

**Figure 1:**
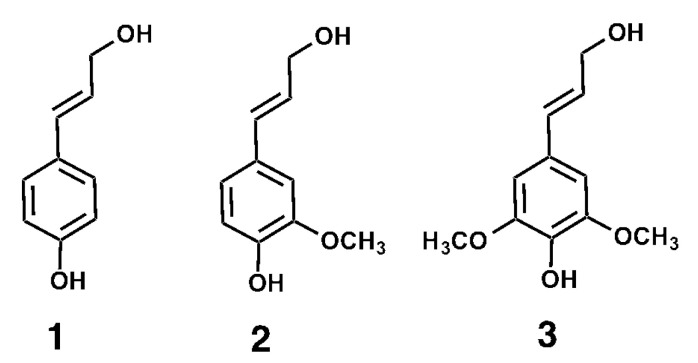
The structure of the monolignols *p*-coumaryl alcohol (**1**), coniferyl alcohol (**2**) and sinapyl alcohol (**3**). These compounds are synthesized in the plant cell and transported to the cell wall where they can undergo oxidative polymerization to form lignin.

In plants, lignin contributes to the structural rigidity of secondary plant cell wall walls, facilitates water transport through the vasculature [6,7], and provides a physical barrier against pests and pathogens [8]. Lignin is formed from the oxidative polymerization of monolignols. The three most common monolignols are *p*-coumaryl alcohol, coniferyl alcohol and sinapyl alcohol (Figure 1). These compounds are synthesized in the plant cell and transported to the cell wall, where they can undergo oxidative polymerization through the action of peroxidases [9] and laccases [10]. Upon incorporation into lignin, the structures derived from the three abovementioned monolignols are referred to as *p*-hydroxyphenyl (H), guaiacyl (G) and syringyl (S) residues, respectively [11,12]. The composition of the lignin differs across plant species. Gymnosperm wood is composed primarily of G residues, whereas hardwood lignin is composed of G and S residues in a 1:2 ratio. Lignin in grasses such as sugarcane, maize and wheat consists of G and S residues in a ratio varying between 2:1 for young tissue and 1:1 for mature tissue, with a small percentage (3–5%) of H residues. The composition of lignin has been shown to vary across plant tissues and cell types [13,14,15].

Lignin is a major industrial by-product of the pulp- and papermaking process as well as of the nascent biorefineries that produce renewable fuels and chemicals from plant biomass. During these industrial processes, the biomass is fractionated in the main polymeric components of the plant biomass: Cellulose, hemicellulosic polysaccharides and lignin [16]. The properties of the lignin that is generated this way vary as a function of the species from which the lignin was obtained (which determines the lignin subunit composition), as well as the chemical and/or physical methods employed during biomass fractionation, some of which rely on the derivatization of lignin with functional groups to enhance solubility. The main sources of industrial lignin are kraft lignin, lignosulfonates, alkali lignin and organosolv lignin, including BioLignin^TM^ [17,18]. Lignin can also be isolated on a small scale using analytical methods, resulting in Klason lignin [19], milled wood lignin (MWL) or Björkman lignin [20], acetylbromide lignin [21] and thioglycolic acid lignin [22].

Industrial lignin has been viewed as a low-value residue that is either burned to generate heat and electricity, used as a source of glue, or additive to concrete or drilling fluids [23,24]. Lignin is, however, the most abundant natural source of aromatic molecules, which makes it attractive for the generation of novel composites and nanomaterials [18] as well as aromatic chemicals [25]. Lignin has also found a use in the biomedical field. Hydrogels, for example, are attractive in tissue engineering because of their similarities to the extracellular matrix and their ability to absorb liquid that is up to a thousand times their dry weight [26]. Additionally, they can protect drugs and small peptides from degradation [27]. Lignin-based nanotubes (LNTs) were synthesized by Caicedo et al. [28], using a sacrificial alumina membrane template. Ten et al. [29] subsequently demonstrated that these lignin nanotubes were able to deliver DNA into human (HeLa) cells without the need for auxiliary agents. Although these LNTs were shown to be less cytotoxic compared to multi-walled carbon nanotubes, their relatively large size, with lengths in the micrometer range, may make them too large to enable the delivery of therapeutic nucleic acids to internal organs in humans or animals, due to the risk of removal by the reticulo-endothelial system or via endosomal degradation [30]. A more recent method to produce lignin nanoparticles (LNPs) relies on solvent exchange. Lievonen et al. [31] described an elegant method in which kraft softwood lignin was dissolved in tetrahydrofuran (THF) and subjected to dialysis against water. As the water entered the dialysis tubing, the lignin started to precipitate, resulting in the formation of spherical LNPs with diameters ranging between 330 and 1300 nm depending on the initial concentration of the lignin.

In this study, we examined whether different sources of lignin (isolation method and plant species) were compatible with the solvent exchange method. Given the variation in lignin structure as a function of plant source and isolation procedure, we hypothesized that the physico-chemical properties and cytotoxicity of the LNPs would vary depending on the source of the lignin. In addition, the ability of these LNPs to bind and deliver DNA was assessed. This was evaluated by using LNPs as a delivery vehicle for a plasmid harboring a reporter gene to transfect A549 lung carcinoma basal epithelial cells in cell culture.

## 2. Materials and Methods

### 2.1. Chemicals 

All chemicals were analytical grade and obtained from Sigma-Aldrich (St. Louis, MO, USA), unless otherwise stated. Linear polyethylenimine (PEI) had an average M_n_ of 2500 and a polydispersity index (PDI) < 1.2. Poly-l-lysine with an average Mw of 84 kDa was purchased from Sigma-Aldrich as a 0.01% solution (cat. no. A-005-M).

### 2.2. Lignin Sources 

Three sources of lignin were used: Wheat BioLignin™ and birch BioLignin™, both kindly provided as powders by Professor Michel Delmas (Compagnie Industrielle de la Matière Végétale (CIMV), Toulouse, France) and thioglycolic acid lignin from poplar. BioLignin™ is an organosolv lignin produced by CIMV on a commercial scale by treating plant biomass with a mixture of acetic acid and formic acid at atmospheric pressure. The resulting raw pulp is filtered, the solvents are removed and the residue is treated with hydrogen peroxide. BioLignin™ is obtained by treating the residue with water, which precipitates the lignin [32,33]. Detailed analysis of wheat BioLignin™ indicated a relatively low molecular weight (1000–1500 g mol^−1^) and linear structure compared to other preparations of lignin [34], which, together with its commercial availability, makes this source of lignin of particular interest.

Thioglycolic acid (TGA) lignin was obtained from greenhouse-grown cuttings of poplar (*Populus deltoides*) based on the method by Bruce and West [35]. Approximately 200 mg dried and ground poplar stem tissue was mixed with 10 mL of 2 M hydrochloric acid (HCl) and 1 mL TGA. The mixture was heated in a water bath at 100 °C for 4 h, followed by centrifugation of the samples at 25,000× *g* for 15 min. at room temperature. The supernatant was discarded, the pellet was washed with 10 mL of ddH_2_O and centrifuged at 25,000× *g* for 15 min. at room temperature. The supernatant was discarded, and the pellet was resuspended in 10 mL 0.5 M NaOH. The samples were gently agitated for 2 days at room temperature to extract the lignin thioglycolate. The samples were then centrifuged at 25,000× *g* for 15 min. at room temperature. The supernatant was collected and 10 mL of ddH_2_O was added, followed by 2 mL concentrated HCl. The lignin was precipitated overnight at 4 °C. The following day, samples were and centrifuged at 25,000× *g* for 15 min. at room temperature. The supernatant was discarded and a lignin pellet was obtained and dried. This source of lignin was selected as an alternative source of hardwood lignin, to examine the effect of the lignin isolation method on the formation of LNPs. TGA lignin contains different functional groups than BioLignin™ and has a greater molecular weight (M_w_ > 10,000 g mol^−1^) [36].

### 2.3. Synthesis of Lignin Nanoparticles 

Poplar TGA lignin, birch and wheat BioLignin™ were used to synthesize lignin nanoparticles using the procedure described by Lievonen et al. [31], with modifications. Lignin was dissolved in THF at concentrations of 0.5, 1, 2, 5, 10 and 20 mg mL^−1^. The dissolved lignin was then filtered through a 0.45 µm syringe filter (GE Healthcare Whatman, Chicago, IL, USA) and introduced into a SnakeSkin dialysis bag (7 kDa MWCO; Thermo Scientific, Waltham, MA, USA), which was then immersed in a beaker with 1.5 L of ddH_2_O and placed on a magnetic stirrer. The water was changed every 8 h over the course of 45 h. A total of three biological replicates were assessed for each concentration. The suspension of lignin nanoparticles was removed from the dialysis bag with a serological pipette and stored at 4 °C. In order to compare the concentration of LNPs among different preparations, a 50 µL sample was mixed with 50 µL THF. The absorbance at 280 nm was measured in a SpectraMax M5 microplate reader (Molecular Devices, San Jose, CA, USA) against a set of lignin standards.

### 2.4. Scanning Electron Microscopy 

The morphology of the lignin nanoparticles was observed using scanning electron microscopy on a FEI Nova 430 (Lausanne, Switzerland) with energy dispersive spectroscopy (EDS) and monochromatic condenser lens (CL) at an accelerating voltage of 10–18 kV. A 10-μL drop containing lignin nanoparticles was deposited on a glass slide (10 mm diameter; Ted Pella Inc. Redding, CA, USA). The water was evaporated at ambient temperature and the sample was sputter coated with a thin layer (10 nm) of Au/Pd before imaging.

### 2.5. Coating of Lignin Nanoparticles with Poly-l-Lysine 

Lignin nanoparticles were coated with poly-l-lysine by mixing 100 µL of a 0.01% (*w*/*v*) poly-l-lysine solution with 900 µL ddH_2_O containing birch or wheat BioLignin™ LNPs, followed by overnight sonication at 80 kHz, 100 W in an Elmasonic P sonicator (Elma Schmidbauer, Singen, Germany). The following day, the suspension was centrifuged in a microcentrifuge at 15,000 rpm for 5 min. and the pellet was washed three times in 2 mL ddH_2_O to remove excess PLL. The pellet was resuspended in 2 mL ddH_2_O.

### 2.6. Determination of Lignin Nanoparticle Size, Zeta Potential and Dispersity 

The size and zeta potential of lignin nanoparticles seeded from Biolignin™ at various starting concentrations and with or without PLL coating were determined using a Malvern Zetasizer Ultra (Malvern Instruments Ltd., Malvern, Worcestershire, UK). For size and zeta potential measurements, each individual sample was measured once. The size of the lignin nanoparticles was determined by dynamic light scattering and the zeta potential by electrophoretic light scattering. The refractive index value of lignin used to determine zeta potential was 1.59 based on Donaldson [37]. The same instrument was also used to determine the dispersity of the LNP preparations, via multi-angle dynamic light scattering (MADLS).

### 2.7. Preparation of Plasmid DNA 

The 5.7 kb plasmid pdsAAV-CB-eGFP [38], containing the enhanced green fluorescent protein (*eGFP*) reporter gene under the control of the chicken beta-actin promoter, was isolated and purified with the Qiagen Plasmid Maxi Purification Kit (Qiagen, Germantown, MD, USA) from an overnight culture of *Escherichia coli* DH5α cells grown in Luria–Bertani (LB) medium at 37 °C. The quality and quantity of the plasmid DNA was analyzed in a NanoVue spectrophotometer (GE Healthcare Life Sciences, Chicago, IL, USA). The purified plasmid was eluted and stored in 10 mM Tris, 1 mM EDTA (TE) buffer pH 8.0 at −20 °C.

### 2.8. Agarose Electrophoretic Mobility Shift Assay 

Complexes of LNPs and plasmid DNA were freshly prepared by mixing 50 ng plasmid DNA with varying amounts of PLL-coated birch LNPs (0.25, 1.25, 2.5, 5, 8.3 and 10 ng) whose original diameter was 286 nm, vortexing for 10 s and incubating for 30 min. at room temperature. Plasmid without LNPs and plasmid mixed with uncoated birch LNPs were included as controls. Agarose gel electrophoresis was carried out in 1× TAE buffer in a 1.0% (*w*/*v*) agarose gel containing GelRed dye at 50 V. The DNA was visualized with a UV transilluminator.

### 2.9. Cell Line Maintenance 

Human lung carcinoma basal epithelial A549 cells (American Type Culture Collection (ATTC) cat. no. CCL-185) were kindly made available by Dr. Maria Zajac-Kaye (UF Department of Anatomy and Cell Biology) and cultured in RPMI-1640 Medium supplemented with 10% fetal bovine serum and 1% (*w*/*v*) penicillin–streptomycin (Sigma R8758). The cells were maintained at 37 °C in humidified air containing 5% CO_2_.

### 2.10. Functionalization of Single-Walled Carbon Nanotubes 

Single walled carbon nanotubes (SWCNT) with a diameter of 0.7–1.3 nm were purchased from Sigma-Aldrich (catalog no. 704113). The SWCNTs were functionalized with amide groups according to the procedure by Ramanathan et al. [39]. Approximately 50 mg SWCNTs were oxidized in a 40 mL of a 3:1 mixture of concentrated sulfuric acid and nitric acid. The mixture was sonicated at 37 kHz for 3 h at 40 °C in an Elmasonic P ultrasonic bath. Following the 3 h sonication, the mixture was added dropwise to 200 mL cold distilled water and then filtered through a 0.45 μm nylon filter (Corning, Corning, NY, USA). The filtrate was washed three times to remove any residual acid and then dried in an oven at 80 °C for 4 h. Next, 20 mg of the dried oxidized SWCNT were dispersed via sonication in 10 mL ethylenediamine, and 1 mg of *N*-[(dimethylamino)-1*H*-1,2,3-triazolo-[4,5-*b*]-pyridin-1-ylmethylene]-*N*-methylmethanaminium hexafluorophosphate *N*-oxide (HATU) was added to the solution to react with the carboxylic acid on the SWCNTs, to produce an ester susceptible to nucleophilic attack by the primary amine group of ethylenediamine. Sonication was continued for an additional 4 h. The product was then added dropwise to 200 mL methanol, filtered through a 0.45 μm nylon filter and washed with excess methanol to remove any residual HATU and ethylenediamine. The functionalized SWCNTs were dried in an oven at 80 °C for 4 h and resuspended in water prior to use

### 2.11. Cytotoxicity Assay 

To evaluate the cytotoxicity of LNPs to A549 lung epithelial cells, 6 × 10^5^ cells were cultured in 100 µL RPMI-1640 medium supplemented with 10% (*w*/*v*) fetal bovine serum and 1% (*w*/*v*) penicillin–streptomycin in a 96-well culture plate for 2 days at 37 °C in 5% CO_2_. To individual cells were added 10 µL containing 6 µg LNPs or PLL-coated LNPs derived from either birch or wheat BioLignin™ of different diameters (based on different starting concentrations of lignin). The LNPs were incubated with the A549 cells at 37 °C and 5% CO_2_ for 4 h. After incubation, the medium was replaced with 100 µL complete RPMI-1640 medium and cells were incubated for another 24 h. Two controls were included with each formulation of LNPs: 0.25 µg amide-functionalized SWCNTs (see prior description) and 50 µg LNTs prepared from poplar TGA lignin [29]. A Cell Count Kit 8 (CCK, Sigma-Aldrich) was used to determine cellular viability. CCK-8 reagent was added to each well and the absorbance at 450 nm was measured using a SpectraMax M5 microplate reader. The absorbance of the non-exposed cells was the reference value for calculating 100% cellular viability. The cytotoxicity assay was performed on three biological replicates.

### 2.12. Delivery of Plasmid DNA Harboring the eGFP Gene into Lung Carcinoma Basal Epithelial Cells 

The transfection efficiency of LNPs was evaluated in A549 lung carcinoma basal epithelial cells, using the *eGFP* reporter gene. A549 cells were seeded in a 12-well plate 24 h prior to the transfection at a density of 6 × 10^5^ per well in complete RPMI-1640 medium. A total of 6 μg each of wheat LNPs with diameters of 160 or 282 nm, and birch LNPs with diameters of 286 or 341 nm, with or without PLL coating, or were mixed with 413 ng plasmid pdsAAV-CB-eGFP in 50 μL serum- and antibiotic-free RPMI-1640 medium and incubated for 1 h at 37 °C. At the time of transfection, the medium in each well was replaced with 300 μL RPMI-1640 medium containing plasmid DNA and LNPs; the cells were then incubated for 4 h. The transfection medium was replaced with 300 μL complete RPMI-1640 medium, and the cells were incubated for an additional 24 h. The following controls were used: plasmid DNA only (413 ng; no LNPs); plasmid DNA mixed with 0.25 µg of amide-functionalized SWCNTs (see prior description); plasmid DNA with 0.5% (*w*/*v*) PEI, a transfection agent commonly used for with cell cultures). Expression of eGFP was analyzed by fluorescence microscopy 24 h after transfection using a fluorescence microscope (DMI 4000B; Leica Microsystems, Wetzlar, Germany) or a confocal microscope (Leica TCS SP5; Wetzlar, Germany). The laser was 405 nm diode UV, the scan speed was 400 Hz, and the numerical aperture 1.4.

### 2.13. eGFP Quantification via ELISA

Transfection of A549 cells was carried out as described above. At 48 h post-transfection, A549 cells were rinsed twice for 10 min. with ice cold phosphate-buffered saline (PBS) and lysed in 300 μL RIPA buffer (1 M Tris.HCl pH 8.0, 5 M NaCl, 1% (*v*/*v*) Nonidet P-40 (surfactant), 10 mM NaF, 0.5 mM EDTA, 10% (*w*/*v*) sodium dodecyl sulfate (SDS), 10% (*v*/*v*) sodium deoxycholate, and 100 μL mL^−1^ protease inhibitor cocktail. The contents of the well were collected in a microcentrifuge tube and centrifuged at 12,000 rpm for 15 min. at 4 °C. The protein concentration in the supernatant was determined using a Pierce BCA protein assay kit (Thermo Fisher, Waltham, MA) in a SpectraMax M5 microplate reader at 595 nm with a standard of bovine serum albumin (BSA). The concentration of eGFP 48 h post transfection was detected using an enzyme-linked immunosorbent assay (ELISA) kit (Cell Biolabs, Inc., San Diego, CA, USA). Purified recombinant GFP standards and cell extracts containing 200 ng total protein were loaded into the wells of a microtiter plate and incubated at 37 °C. The plate was washed three times with 1× wash buffer (provided in the kit), followed by the application of biotinylated anti-GFP antibody (provided in the kit; 1:1000 dilution). The plate was then incubated at room temperature for 2 h. The plate was washed three times using 1× wash buffer, followed by the application of streptavidin-enzyme conjugate secondary antibody (provided in the kit; 1:2000 dilution). The plate was incubated at room temperature for 1 h, washed three times with 1× wash buffer and incubated with the substrate solution (provided in the kit) for 25 min. at room temperature. The reaction was stopped by adding stop solution (provided as part of the kit) and the absorbance was read at 450 nm in a SpectraMax M5 microplate reader. The concentration of eGFP was determined using a standard curve based recombinant GFP standards provided with the kit.

## 3. Results

### 3.1. Relationship between Initial Lignin Concentration and the Size of the Nanoparticles

**Figure 2:**
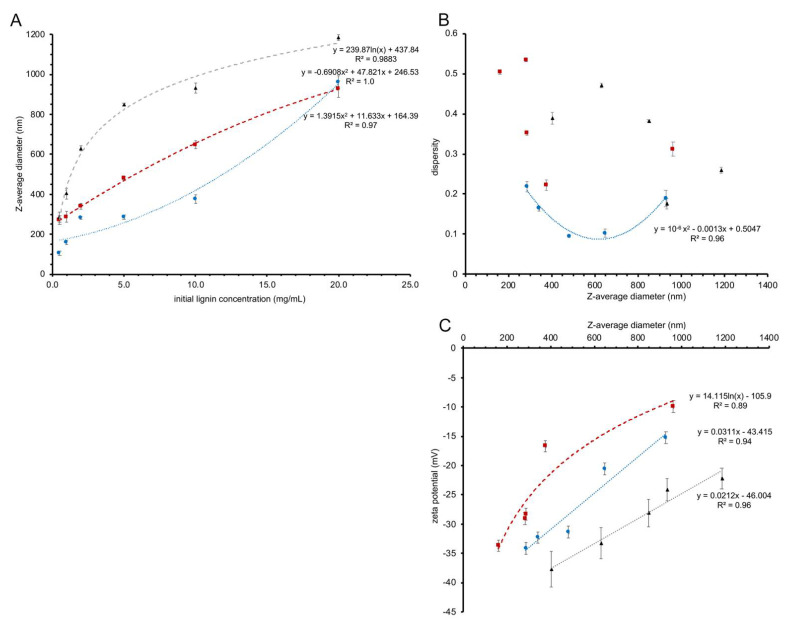
(**A**) Z-average size of nanoparticles made from birch BioLignin™ (red squares), wheat BioLignin™ (blue circles) and poplar TGA lignin (black triangles) as a function of the concentration of lignin at the start of the solvent exchange. The trend lines represent the best-fit polynomial (birch and wheat LNPs) or logarithmic function (poplar LNPs) for which the function and R^2^ value are displayed. (**B**) Dispersity of nanoparticles made from birch BioLignin™ (red squares), wheat BioLignin™ (blue circles) and poplar TGA lignin (black triangles) as a function of their diameter, determined via dynamic light scattering. The trend line represents the best-fit polynomial, which could only be obtained for the wheat LNPs. (**C**) Zeta potential of nanoparticles formed from birch BioLignin™ (red squares), wheat BioLignin™ (blue circles) and poplar TGA lignin (black triangles) as a function of their diameter, determined via electrophoretic light scattering. The trend lines represent the best-fit polynomial (birch and poplar LNPs) or logarithmic function (wheat LNPs) for which the function and R^2^ value are displayed. In all three graphs the error bars represent the standard deviation based on three replicates.

Three different sources of lignin—birch BioLignin™, wheat BioLignin™ and poplar TGA lignin—were used to obtain LNPs using the solvent exchange procedure and to examine the relationship between the initial concentration of lignin and particle size. Dynamic light scattering using a Zetasizer instrument was used to determine the average diameter of LNPs formed with initial concentrations of 0.5, 1.0, 2.0, 5.0, 10 and 20 mg mL^−1^ lignin. With all three lignin sources the diameter of the LNPs depended on the initial concentration of lignin, whereby the diameter increased with the starting concentration (Figure 2A). A higher initial concentration of lignin in the dialysis bag will tend to favor aggregation of lignin molecules via hydrophobic interactions during the solvent exchange, when the hydrophobic lignin is exposed to increasing concentrations of water. Conversely, at lower initial concentrations, there are fewer opportunities for lignin molecules to aggregate, resulting in LNPs with a smaller diameter. Although the average diameter increases with the starting concentration of the lignin for all three sources, the trend lines fitted through the data points (Figure 2A) have different shapes, suggesting that the aggregation dynamics vary as a function of lignin source. For example, LNPs formed from birch and wheat BioLignin™ at a concentration of 20 mg mL^−1^ had similar Z-average diameters; however, at lower concentrations, the diameters of LNPs formed from wheat BioLignin™ were smaller, indicating weaker interactions at the nanoscale range compared to birch BioLignin™, possibly due to greater solubility in water.

### 3.2. Characterization of Lignin Nanoparticles

**Figure 3:**
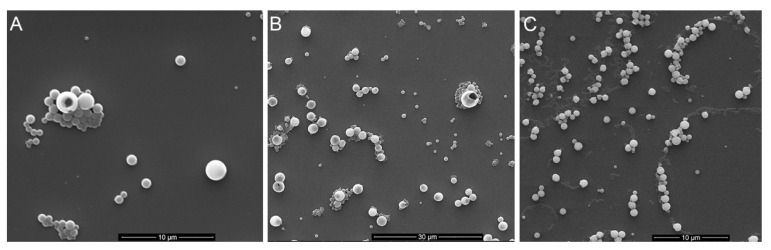
Scanning electron micrographs showing LNPs made from: (**A**) birch BioLignin™ (286 nm diameter; 18 kV; 5000× magnification); (**B**) wheat BioLignin™ (282 nm diameter; 18 kV; 1975× magnification); (**C**) Poplar TGA lignin (322 nm diameter; 10 kV; 4000× magnification). The scale bars in the bottom of the images indicate the size.

A Zetasizer instrument was also used to determine the dispersity of the different formulations of LNPs. There was no obvious relationship between the size of the LNPs and their dispersity (Figure 2B; Supplemental Figure S1). The dispersity values for LNPs produced from birch BioLignin™ were overall lower than for LNPs produced with the other two sources of lignin, implying a more uniform distribution. This was confirmed with scanning electron microscopy (Figure 3). The LNPs are spherical in shape, but it is apparent that there are particles of varying diameters in each of the preparations of LNPs, with the diameters of the smaller particles being most similar to the Z-average diameters measured with the Zetasizer instrument (Supplemental Figure S2). The variation in diameter is smallest among the wheat LNPs. The observed variation in diameter is likely the result of a non-uniform distribution of the molecular weights of the different lignin preparations, combined with the effect of gravity during the dialysis, which will increase the proportion of larger particles in the lower portion of the dialysis tubing.

The LNPs synthesized from all three sources of lignin displayed negative zeta potentials. Figure 2C shows the relationship between the Z-average diameter of the LNPs (based on Figure 2A) and their zeta potential. In all cases, the zeta potential became less negative as the size of the nanoparticles increased. The LNPs synthesized from wheat Bio Lignin™ had less negative zeta potentials than the LNPs synthesized from birch BioLignin™ with similar diameters, which likely reflects differences in the structure of the lignin obtained from the two plant species. The LNPs synthesized from poplar TGA consistently had more negative zeta potentials than similar-sized LNPs produced from the other two lignin sources. Since birch and poplar are both hardwood species, their lignin subunit composition will be similar, and the difference in zeta potential is likely due to the different lignin extraction methods (molecular weight and functional groups that were introduced).

The aggregation of lignin during the solvent exchange is driven by hydrophobic interactions among the lignin fragments as the solvent becomes more polar. However, all three lignin sources contain acid moieties (from acetic and formic acid for the wheat and birch BioLignin™ and from thioglycolic acid for the poplar lignin) that were introduced during the extraction procedure and that are assumed to be distributed uniformly within the lignin fragments. While the hydrophobic domains of the lignin will be in the LNP’s core, these deprotonated acid moieties will interact with water on the surface of the LNPs and cause the negative zeta potential. As the LNPs continue to grow while the concentration of water increases, additional lignin molecules will be more prone to being added to the outside of an LNP, because rearrangement of the hydrophobic core is not energetically favorable due to the (temporary) exposure to the water molecules that would be necessary. These newly added fragments will shield some of the existing negative charges on the perimeter. Consequently, small particles are expected to display a more uniform and denser distribution of negative charges on the outside. The larger particles have proportionally fewer acid groups on their surface, leading to a less negative zeta potential. The smaller LNPs with their more negative zeta potentials will be more stable in suspension (less likely to aggregate) due to the combination of greater repulsion between individual particles and enhanced Brownian motion.

Since DNA has a negative charge at neutral pH due to the presence of phosphate groups in its backbone, it was unlikely that DNA would associate with LNPs displaying negative zeta potentials. After preliminary experiments involving coating LNPs with low-molecular-weight chitosan were not successful (data not shown), coating of LNPs with the cationic polymer poly-l-lysine (PLL) was explored, based on the successful use of PLL in prior gene therapy studies [40,41]. To examine the effect and efficacy of PLL coating, the zeta potential and diameter of LNPs synthesized from birch BioLignin™ at a starting concentration of 2 mg mL^−1^ were determined. The diameter increased from 341 to 357 nm and the zeta potential changed from −29.5 to + 43.2 mV. Under the conditions used, it was not possible to obtain zeta potential readings for PLL-coated LNPs with greater starting diameters, likely as a result of multiple scattering and particle-particle interactions.

### 3.3. Electrophoretic Mobility Shift Assay (EMSA)

**Figure 4:**
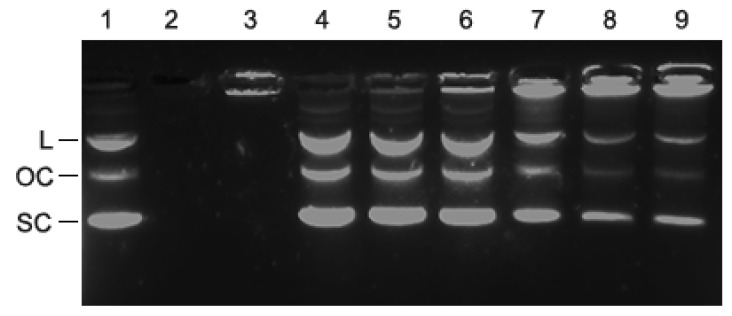
An agarose gel stained with GelRed dye after electrophoresis of plasmid DNA that had been incubated with increasing amounts of PLL-coated birch LNPs (286 nm diameter) shows that the DNA and the PLL-coated LNPs interact. (**1**) 50 ng plasmid DNA (positive control). The three DNA bands represent the three configurations of the plasmids as indicated on the left (linear (L), open circle (OC), supercoiled (SC)), that migrate through the gel at different velocities; (**2**) 20 ng uncoated birch LNPs, no plasmid DNA; (negative control); (**3**) 20 ng PLL + 50 ng plasmid DNA (negative control); (**4**) 0.25 ng PLL-coated birch LNPs with 50 ng plasmid DNA (1:200 ratio); (**5**) 1.25 ng PLL-coated LNPs with 50 ng plasmid DNA (1:40); (**6**) 2.5 ng PLL-coated birch LNPs with 50 ng plasmid DNA (1:20 ratio); (**7**) 5.0 ng PLL-coated birch LNPs with 50 ng plasmid DNA (1:10 ratio); (**8**) 8.3 ng PLL-coated birch LNPs with 50 ng plasmid DNA (1:6 ratio); (**9**) 10 ng PLL-coated birch LNPs with 50 ng plasmid DNA (1:5 ratio).

Agarose gel electrophoresis was performed to determine whether PLL-coated LNPs were able to interact with DNA. Plasmid DNA bound to PLL-coated LNPs was hypothesized to not migrate through the gel as a result of the large size and/or neutralization of the negative charge of the DNA by the PLL. A fixed amount of 50 ng plasmid DNA was loaded on the well by itself, or mixed with PLL, or mixed with increasing amounts of PLL-coated LNPs. As can be observed in Figure 4, the combination of PLL+DNA (lane 3) prevented the DNA from migrating into the gel, because the positively charged PLL shielded the negative charge on the DNA. The increasing intensity of fluorescence in the wells corresponding to DNA bound to PLL-coated LNPs and the decreasing intensity of the DNA that migrated into the gel (lanes 4–9), indicate that PLL-coated LNPs retarded the migration of plasmid DNA compared to plasmid DNA control (lane 1). The fact that there is a dosage-dependent effect of the PLL-coated LNPs suggests an association between the DNA and the PLL-coated LNPs.

### 3.4. Cytotoxicity of LNPs as a Function of Size and Lignin Source

**Figure 5:**
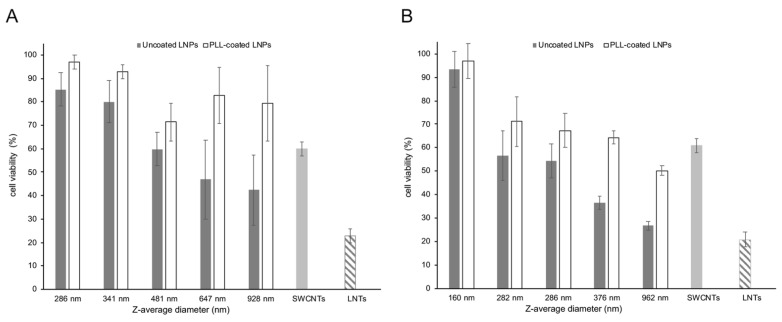
Cytotoxicity of LNPs of different sources and diameters determined with the Cell Count Kit-8 on A549 cells. The vertical axis displays cell viability (%). (**A**) Uncoated and PLL-coated birch BioLignin™ nanoparticles. (**B**) Uncoated and PLL-coated wheat BioLignin™ nanoparticles. For comparison, SWCNTs (light gray bars) and LNTs prepared from poplar TGA lignin (hatched bars) were included. The error bars represent the standard deviation of three replications.

One of the concerns over the use of nanoparticles as delivery vehicles for therapeutic nucleic acids is their cytotoxicity. In order to assess the cytotoxicity of LNPs on A549 cells, the proportion of live cells remaining following exposure of a fixed amount of coated and uncoated LNPs of different sizes were determined. This assay, as well as the subsequent transfections (Section 3.4), were performed with the two sources of BioLignin™, as being more representative of industrial lignin than the thioglycolic acid lignin. In addition, this made it possible to assess the impact of lignin subunit composition (hardwood versus grass lignin). As shown in Figure 5A (birch BioLignin™) and Figure 5B (wheat BioLignin™), as the size of the uncoated LNPs increased, the overall viability of A549 cells decreased. The birch LNPs had a somewhat lower cytotoxicity than the wheat LNPs. This is apparent from the data for LNPs of similar diameter: when cells were exposed to birch LNPs with a Z-average diameter of 286 nm, the viability was 85%, versus 54% for the wheat LNPs of the same Z-average diameter. Similarly, the cell viability after exposure to birch LNPs with a diameter of 928 nm was 42%, versus 27% for the wheat LNPs with a Z-average diameter of 962 nm. The cell viability was overall greater when PLL-coated LNPs were used, for both birch and wheat LNPs. The smallest size PLL-coated LNPs of either type did in fact not display any cytotoxicity. The effect of the PLL coating was greater for the LNPs with the larger diameters. The variance in cell viability was greater with the birch LNPs compared to the wheat LNPs, which is likely the result of the higher dispersity values of the former (see Figure 2B), indicative of a greater range in diameter and hence, in cytotoxicity.

Amide-functionalized SWCNTs and LNTs prepared from poplar TGA lignin were included in the cytotoxicity assay as comparisons. To accommodate the 20–100-fold smaller size of SWCNTs, a smaller amount (0.25 μg) was used than for the LNPs (6 μg). Similarly, for the larger-sized LNTs, an eight-fold greater amount (50 μg) was used. The controls were not coated with PLL to reflect their use in prior transfection experiments. Under these conditions, the SWCNTs appear to have a similar cytotoxicity as the LNPs, whereas the cytotoxicity of the LNTs is somewhat higher than the LNPs. This is likely the result of their larger size, given that the cytotoxicity of the LNPs also displayed a size dependency.

### 3.5. Transfection of A549 Lung Carcinoma Basal Epithelial Cell with LNPs

A549 lung carcinoma basal epithelial cells were transfected to assess the ability of LNPs to deliver pds-AAV-CB-EGFP. The transfection efficiency was evaluated by fluorescence microscopy, confocal microscopy and ELISA. A549 cells were exposed to PLL-coated LNPs, PEI, each complexed with the plasmid pds-AAV-CB-eGFP which encodes the enhanced green fluorescent protein (eGFP). The transfections with PEI and with derivatized SWCNTs were used as positive controls. Negative controls consisted of cells that received no treatment, cells that had been incubated with just DNA, and cells that had been incubated with a mixture of DNA and PLL.

**Figure 6:**
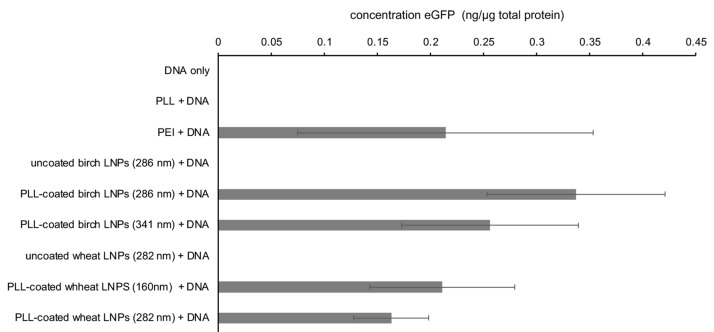
Concentration of eGFP protein in cell lystate determined via ELISA 48 h post transfection of 5 × 10^6^ A549 cells with pdsAAV-CB-eGFP plasmid DNA delivered via the different means displayed along the vertical axis.

The results from the ELISA (Figure 6) indicated that the assay conditions were conducive to transfection using PEI as a positive control, whereas no fluorescence was observed for the negative controls (DNA only; DNA and PLL). Based on the presence of eGFP in the cells that had been transfected with PLL-coated birch and wheat BioLignin™ nanoparticles of two different sizes (birch, 286 and 341 nm and wheat, 160 and 282 nm) were able to deliver DNA into A549 cells with similar efficacy as PEI. The efficacy of the LNP-mediated transfection mirrors the cytotoxicity data (Figure 5), with PLL-coated birch LNPs outperforming PLL-coated wheat LNPs, and LNPs with smaller diameters outperforming LNPs with larger diameters. However, due to the substantial variance, these are mere trends and will require more detailed investigation.

**Figure 7:**
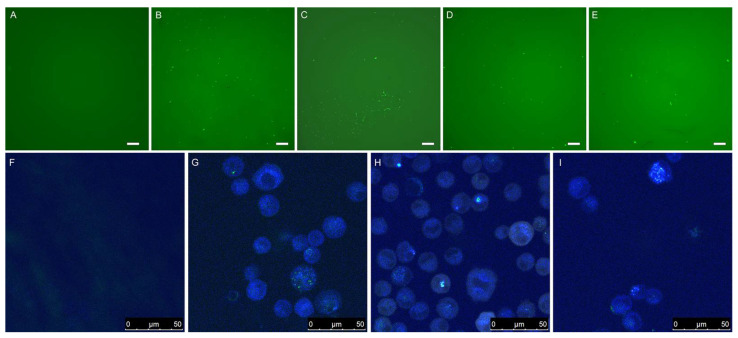
The top five images were taken with a fluorescence microscope at 40× magnification and show A549 cells 48 h post transfection with pdsAAV-CB-eGFP. The white scale bar in the bottom right corner indicates 200 μm. (**A**) Plasmid DNA control; (**B**) PEI + DNA; (**C**) SWCNTs + DNA; (**D**) PLL-coated birch LNPs (286 nm diameter) + DNA; (**E**) PLL-coated wheat LNPs (282 nm diameter) + DNA. The bottom four images were taken with a confocal microscope and show the results from a separate transfection experiment. (**F**) Plasmid DNA control; (**G**) Functionalized single-walled carbon nanotubes + DNA; (**H**) PLL-coated birch LNPs (286 nm diameter); (**I**) PLL-coated wheat LNPs (282 nm diameter) + DNA. Scan speed was 400 Hz, numerical aperture was 1.4 and the laser was 405 nm diode UV. The scale bar in the bottom right corner indicates the size.

Fluorescence microscopy (Figure 7A–E) and confocal microscopy (Figure 7F–I) were used to visualize expression of eGFP in A549 cells 48 h after transfection. No fluorescence was observed with negative controls consisting of plasmid DNA without carriers, whereas PLL-coated birch and wheat LNPs were able to deliver DNA inside the cells, similar to what was observed with the positive controls (Figure 7B,C,G).

## 4. Discussion

The solvent exchange method was used to generate LNPs from softwood kraft lignin by Lievonen et al. [31]. They reported that the diameter of the LNPs varied as a function of the initial concentration of lignin. In this study, we have shown that the solvent exchange method is also compatible with other sources of lignin (different plant species and different lignin isolation methods). The range in diameter we reported (160–1194 nm) was shifted to slightly lower values than the 300–1300 nm range reported by Lievonen et al. [31], which is of relevance given the greater cytotoxicity of LNPs with larger diameters. The combined data show that the source of the lignin affects the aggregation dynamics, which is likely a function of the structure of the lignin, specifically lignin subunit composition, the molecular weight, and the nature of the moieties introduced during the extraction method. Although a higher starting concentration of lignin consistently results in LNPs with a larger diameter, it will be necessary to determine the exact relationship between starting concentration and LNP diameter for each additional source of lignin that is being considered. A further factor that will need to be investigated is whether the process can be scaled up. Specifically, if the goal is to produce larger batches of LNPs with the use of larger dialysis systems, the aggregation dynamics may change due to the larger volume of solvents that needs to be exchanged.

Related to the aggregation dynamics is the dispersity. Although the initial concentration of the lignin determined the average size (diameter) of the LNPs, the LNPs that resulted from our three sources of lignin as well as LNPs from kraft lignin made by Lievonen et al. [31] clearly displayed a range in size that was independent of the initial lignin concentration. Instead, this may be the result of solvent gradients and/or gravity-based density gradients inside the dialysis tubing. Another factor may be the molecular weight distribution of the lignin itself, although the reported narrow molecular weight distribution makes this less plausible in the case of wheat BioLignin™ [34]. These are factors that will also need to be evaluated and controlled during future scale-up experiments, as medical applications tend to require (nano)materials that have well-defined and consistent properties. It is likely that the dispersity contributes to the fairly large variances observed in the cytotoxicity data, especially for the birch LNPs.

The three sources of lignin we evaluated in this study contained negatively charged groups that were introduced during the extraction procedures and that were responsible for the negative zeta potentials. The zeta potential itself was dependent on the source of the lignin and on the particle diameter, with smaller particles displaying more negative zeta potentials and thus greater stability in suspension. The zeta potentials of these LNPs are less negative than the values reported by Lievonen et al. [31] for LNPs made from kraft lignin (−60 mV). These differences are likely due to the introduction of more negatively charged moieties during the initial lignin extraction. The observation that the zeta potential increased (i.e., became less negative) as the diameter of the LNPs increased was also consistent with the data reported by Lievonen et al. [31].

Coating the particles with positively charged PLL was necessary to change the zeta potential and enable interaction with negatively charged DNA. Although the coating with PLL represents an additional step to make the LNPs compatible with DNA, it is not difficult to accomplish, does not contribute substantially to the cost, and has as an important additional benefit that the cytotoxicity of the LNPs is reduced, as shown in Figure 5. Although PLL and PLL-containing co-polymers have been used in prior gene therapy experiments because of its efficiency in condensing DNA [40,41], PLL, has been reported to be cytotoxic due to its ability to induce cell death via a caspase cascade [42]. Consequently, exploration of alternative cationic polymers may be worthwhile.

The need for PLL-coating to enable the interaction with DNA represents a contrast with lignin nanotubes (LNTs) synthesized in a sacrificial alumina template [29]. DNA appeared to associate spontaneously with the LNTs and the LNTs then served as vehicles for DNA delivery. This implies the surface charge of the LNTs differs from the surface charge of the LNPs, which is likely the result of the differences in how the external surfaces of the two structures were formed. In the case of the solvent exchange procedure used to form LNPs, the hydrophobic domains of the lignin were internalized, exposing the charged groups to the outside of the LNPs, and no additional chemical reactions occurred. The LNT synthesis, in contrast, relies on a Schiff’s base reaction between amine moieties that had been introduced to the pore walls of the membrane and aldehyde end groups in the lignin. The LNTs were subsequently released by dissolving the membrane in 5% (*v*/*v*) phosphoric acid [28], which may have further modified the external surface.

LNTs synthesized from specifically alkaline lignin were able to penetrate the nuclei of HeLa cells, as evidenced by confocal microscopy [29]. At the highest concentrations tested, these LNTs were also the most cytotoxic to HeLa cells among the LNTs investigated by Ten et al. [29]. The confocal images shown in Figure 7 do not show evidence of nuclear localization, but show a fairly disperse distribution, which is in fact why the cells are visible in the first place. If the goal is to use LNPs to disrupt cancer cells, purely based on the cytotoxicity data presented in Figure 5, this may be best accomplished with uncoated LNPs of larger diameters. It may be possible to develop functionalized LNPs that are able to penetrate cell nuclei, but this will require exploration of different types of lignin, i.e., plant species and extraction method. If, on the other hand, the goal is to use LNPs for the delivery of therapeutic DNA, LNPs with small diameters are preferred due to their low cytotoxicity and greater likelihood of evading the reticulo-endothelial system and endosomal degradation.

This cell targeting approach assumes, however, that cytotoxicity is fairly consistent across cell types. The data presented in Figure 5 suggest that may not be the case. The cytotoxicity of LNTs prepared from poplar TGA lignin had been reported to be low in HeLa cells (>80% viability) [29], whereas the data presented in Figure 5 indicate a higher level of cytotoxicity (23% cell viability) in A549 cells. The functionalized SWCNTs also behaved differently in A549 cells than was expected based on the low cytotoxicity (90% cell viability) of similar SWCNTs reported by Pantarrotto et al. [43] in HeLa cells.

In summary, we have shown that the solvent exchange procedure can be used for the synthesis of LNPs with tunable properties that are a function of the initial concentration of the lignin and the lignin source. The ability to use PLL-coated LNPs as vehicles for the delivery of DNA into human cells makes them of interest for therapeutic purposes. In light of such applications, future experiments should focus on the following aspects: (**1**) scaling up LNP synthesis to enable commercial production while reducing the dispersity to enable obtain greater uniformity and more predictable behavior of the LNPs; (**2**) investigating mechanisms to covalently bind cationic polymers to LNPs; (**3**) assessing the stability of native LNPs and LNPs coated with cationic polymers during long-term storage; (**4**) assessing the stability and fate of LNPs coated with cationic polymers in vivo (cell cultures, then mice); (**5**) assessing the in vivo stability and fate of DNA complexed with LNPs coated with cationic polymers to assess the potential for functionalizing LNPs to enable smart delivery to target cells, tissues or organs; (**6**) assessing the immunogenicity of LNPs. Given that fruits and vegetables in the human diet contain lignin, there is no *a priori* reason to anticipate an immune response to native lignin. However, since some sources of industrial lignin contain various chemical modifications, the resulting LNPs may trigger an immune response.

The prospect of adding lignin nanoparticles with tunable physico-chemical properties and low cytotoxicity to the gene therapy toolbox and potentially expand treatment options merit these subsequent investigations.

## Data Availability

The datasets generated and analyzed during the current study are available from the corresponding author on reasonable request.

## Supplementary Materials

The following supporting information can be downloaded at: https://www.mdpi.com/article/10.3390/ma15010303/s1, Figure S1: Histograms displaying the size distributions of the LNPs based on the dynamic light scattering analysis shown in Figure 2B; Figure S2: Histograms displaying the size distributions of the LNPs based on SEM images in Figure 3.
