# Supplementary material for: Delivery of DNA into Human Cells by Functionalized Lignin Nanoparticles
Source: Materials (Basel). 2022 Jan 1;15(1):303. doi: 10.3390/ma15010303 (PMC8745861; doi:10.3390/ma15010303)
Supplement: Supplementary file 1 [file materials-15-00303-s001.zip › materials-1500380-supplementary.pdf]

materials

Supplemental Information

Delivery of DNA into Human Cells by Functionalized Lignin Nanoparticles

Michael K. Riley II [1] and Wilfred Vermerris [2,3,4,*]

[1] Graduate Program in Plant Molecular & Cellular Biology, University of Florida, Gainesville, FL 32610, USA; mike.riley350@gmail.com
[2] Department of Microbiology & Cell Science, University of Florida, Gainesville, FL 32610, USA
[3] UF Genetics Institute, University of Florida, Gainesville, FL 32610, USA
[4] Florida Center for Renewable Chemicals and Fuels, University of Florida, Gainesville, FL 32610, USA
* Correspondence: wev@ufl.edu

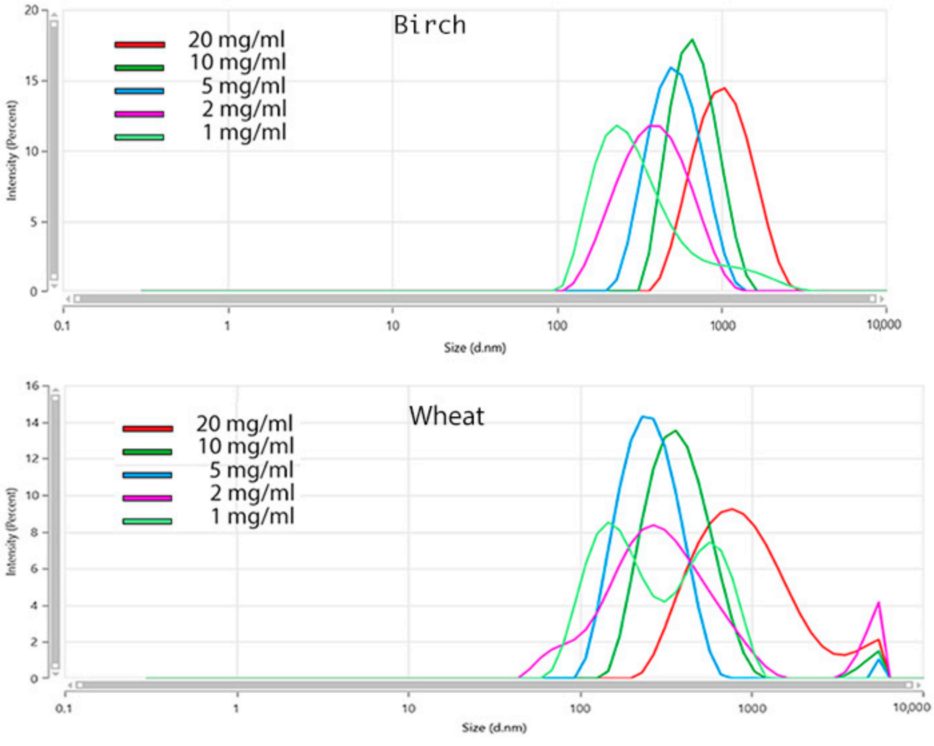

Figure S1. Histograms displaying the size distributions of the LNPs prepared from birch BioLignin™ (top) and wheat BioLignin™, based on the dynamic light scattering analysis shown in Figure 2B. Data obtained with the ZS Xplore software (Malvern Panalytical).

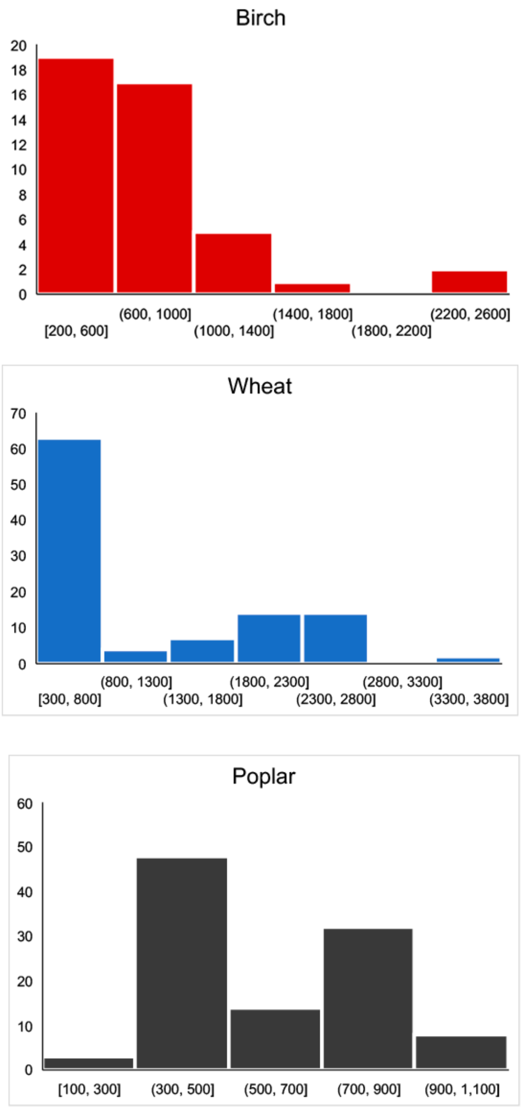

Figure S2. Histograms displaying the distributions of diameters of the LNPs prepared from birch BioLignin™ (top), wheat BioLignin™ (center) and poplar TGA lignin (bottom) based on analysis of the SEM images in Figure 3. The horizontal scale displays the diameter range for each bin in nanometer.
